# Paying Wards at Zurich

**Published:** 1906-03-24

**Authors:** 


### PAYING WARDS AT ZURICH.

For many years the question of accommodation for those who are able and anxious to pay, but who cannot afford the very high fees which the nursing homes of London are obliged (partly owing to the situation in which they are forced to locate themselves) to charge, has been of much interest to me, and recently when visiting Zurich I tried to learn a little of the system which there appears to work very well. My time there was short and my knowledge of the German language nil, otherwise I might have had more information to give; but now that the *Times* has stirred up some public interest in the question, possibly the little information I did glean may be of some help to those who are trying to work out a suitable scheme for London.

In the first place, no one of the middle class, and very few of the upper class, in Zurich would arrange to have an operation performed at home; they fully realise the advantages that can only be obtained in a hospital. In the second place, the doctors (or professors as they are called out there) are very averse to performing any serious operation in a private house; and, as they can take fees from patients in the hospitals, they naturally prefer to do their operations there. In the third place, none of the hospitals (except those which seem to correspond to our workhouse infirmaries) appear to accept any patients without payment, so that in entering a hospital there is no feeling of accepting charity.

The payment varies a great deal according to which hospital is chosen and to the circumstances of the patients. I found that some were paying a franc a day for a bed in a general ward, and others, for more elaborate and private accommodation, up to twelve or twenty francs a day. For a small extra charge they could have a special nurse if they wished or if the doctor considered it necessary. On first looking at this scheme one might think it hard that these fine hospitals (by insisting upon some payment) should be closed to the very poor, who, of course, cannot pay even a franc a day, but this is not so. If a man (or woman) is ill and needs hospital treatment, but cannot pay for it, he has to apply to a society which seems to correspond to our Charity Organisation Society. Inquiries are then made, and if the patient's statements are found to be correct, he is immediately admitted to the hospital that is most suitable for his case, and the payments are made to the hospital for him from the funds of this society, on the basis of the lowest fee that that particular hospital accepts.

This applies to the maternity and to the children's hospitals, as well as to the general hospitals.

At the present moment there are doubtless many hundreds (if not thousands) of patients in the London hospitals who could without any difficulty, and in most cases would gladly, pay at least a shilling a day, and with many of them five shillings a day would not be grumbled at. It seems strange that they cannot be allowed and encouraged to do so; and yet if the individual hospitals do allow any patients to pay, they are immediately descended upon by the Committee of the King's Fund, and the help from that source is withdrawn. Would it not be possible for the hospitals, now in grand and complete working order, to open their doors to those who can pay; and for the Committee of the King's Fund or some other organisation (such as the Charity Organisation Society) to constitute a board of inquiry, with officers in every large hospital and at other centres, to find out the circumstances of those who say they cannot pay, and, if necessary, to pay for them from the funds of the society? For the London hospitals—with all the elaborate and expensive fittings that are considered so essential with the present-day surgery—to be self-supporting is, I fear, too much to hope, but there can be no doubt that if all who could afford it paid what they could the necessity for such constant "special appeals" would be largely done away with, and then the managers might be able to turn their attention to providing more and better accommodation for the higher class patients. And if, during the next few years, the money saved in the pockets of the rich and charitable givers should be expended in increasing the number of really first-rate hospitals, surely we should soon get sufficient beds to meet the ever-increasing demand for them. I have some Swiss friends resident in Zurich, and should be glad to obtain any further particulars that may be desired of the system at work there if it would be of any help to those who are interested in this very important question of the day.

